# A novel role for methyl cysteinate, a cysteine derivative, in cesium accumulation in *Arabidopsis thaliana*

**DOI:** 10.1038/srep43170

**Published:** 2017-02-23

**Authors:** Eri Adams, Takae Miyazaki, Aya Hayaishi-Satoh, Minwoo Han, Miyako Kusano, Himanshu Khandelia, Kazuki Saito, Ryoung Shin

**Affiliations:** 1 RIKEN Center for Sustainable Resource Science, 1-7-22 Suehiro-cho, Tsurumi-ku, Yokohama, Kanagawa 230-0045, Japan; 2 MEMPHYS, Center for BioMembrane Physics, University of Southern Denmark, Campusvej 55, Odense M 5230, Denmark; 3 Graduate School of Life and Environmental Sciences, University of Tsukuba, 1-1-1 Ten-noudai, Tsukuba, Ibaraki 305-8572, Japan; 4 Graduate School of Pharmaceutical Sciences, Chiba University, Chiba 260-8675, Japan

## Abstract

Phytoaccumulation is a technique to extract metals from soil utilising ability of plants. Cesium is a valuable metal while radioactive isotopes of cesium can be hazardous. In order to establish a more efficient phytoaccumulation system, small molecules which promote plants to accumulate cesium were investigated. Through chemical library screening, 14 chemicals were isolated as ‘cesium accumulators’ in *Arabidopsis thaliana*. Of those, methyl cysteinate, a derivative of cysteine, was found to function within the plant to accumulate externally supplemented cesium. Moreover, metabolite profiling demonstrated that cesium treatment increased cysteine levels in *Arabidopsis*. The cesium accumulation effect was not observed for other cysteine derivatives or amino acids on the cysteine metabolic pathway tested. Our results suggest that methyl cysteinate, potentially metabolised from cysteine, binds with cesium on the surface of the roots or inside plant cells and improve phytoaccumulation.

The recovery of high-value or toxic metals from soil and water attracts considerable attention from engineers, economists and environmentalists. Biological methods especially, which are generally cost effective and eco-friendly, have recently gained in popularity as an alternative to the conventional physical and chemical approaches to capture metals from soil or water. Biological methods can be divided largely into two categories: biosorption and bioaccumulation (or bioextraction). Biosorption uses materials of a biological origin which is generally pre-treated with chemicals to optimise the surface physicochemical properties for adsorption whereas bioaccumulation uses the ability of living organisms to naturally absorb the metal of interest^1^. Bioaccumulation by plants, more specifically termed as phytoaccumulation, is extremely useful where a valuable/toxic metal is to be directly extracted from soil without disrupting the soil structure but the most common downside with phytoaccumulation is low uptake efficiency. Therefore, it would be beneficial to develop methods which enable improved efficiency in phytoaccumulation of the target metal. Of the many commercial and high-value metals, cesium exists at relatively low levels of approximately 3 ppm in the Earth’s crust^2^. Cesium is utilised widely industrially for example in the production of drilling fluids and in the manufacture of atomic clocks. Radiocesium-137 also has medical, hydrological and industrial applications. Currently, mining of pollucite ore is the major source of cesium production but mining is conducted in only very few places and only on a small scale. Although cesium reserves are said to be sufficient for consumption at the present rate, since cesium is not recycled, it is vulnerable to depletion in the long term^3^. Additionally, cesium-137, the radioactive isotope derived from nuclear waste and fallout, presents a serious health risk and environmental threat such that improved phytoaccumulation efficiency of cesium offers advantages in the light of phytoremediation also. Many plant species have been tested in the search for ‘hyperaccumulator plants’ of cesium, however most of the species tested suffer low absorption efficiency for practical application in the soil^4^,^5^,^6^,^7^,^8^,^9^,^10^,^11^,^12^. Various factors have been investigated and suggested as positive regulators of cesium accumulation in plants. For example, inoculation with root-associated microorganisms has been shown to improve cesium accumulation in plants^13^,^14^,^15^,^16^. Some researchers have reported that an arbuscular mycorrhizal (AM) fungi improve cesium phytoaccumulation efficiency^17^,^18^, while others have contradicted this^19^,^20^, and there is an ongoing debate on the effect of cesium on colonisation of AM fungi^21^. Others have proposed soil amendment with small molecules such as ethylene diamine tetraacetic acid (EDTA) and organic acids as an option to increase cesium accumulation in plants^22^,^23^. The use of ammonium has given contradicting results in respect of cesium accumulation in various plant species and soil types^24^,^25^,^26^. Other factors such as elevated CO_2_ and decreasing concentrations of potassium, sodium and calcium have also been reported as contributing to increased cesium phytoaccumulation^27^,^28^,^29^,^30^.

Here we investigated ways to improve phytoaccumulation efficiency of cesium through the aid of small chemical compounds. In previously published research, we successfully isolated a chemical compound, CsTolen A, through chemical library screening and found that this reduced cesium accumulation by inhibiting cesium entry into plant cells^31^. In this current study, a chemical library composing 10,000 synthetic organic compounds was screened for those which promote cesium accumulation in *Arabidopsis thaliana*. Fourteen chemicals were isolated as cesium accumulators and of those one was characterised as a cysteine derivative, methyl cysteinate. There was a tendency of cysteine to accumulate cesium in plants but the effect was limited compared to that of methyl cysteinate. By contrast, metabolite profiling revealed that internal cysteine concentrations increased upon cesium treatment. Other cysteine derivatives and amino acids on the cysteine biosynthesis pathway, on the other hand, did not show a similar cesium accumulation effect. It has been suggested from our results that external cesium increases internal cysteine levels in plants and is potentially metabolised into methyl cysteinate which contributes to cesium accumulation through binding with cesium.

## Results

### Screening for cesium accumulator chemicals

**Figure 1:**
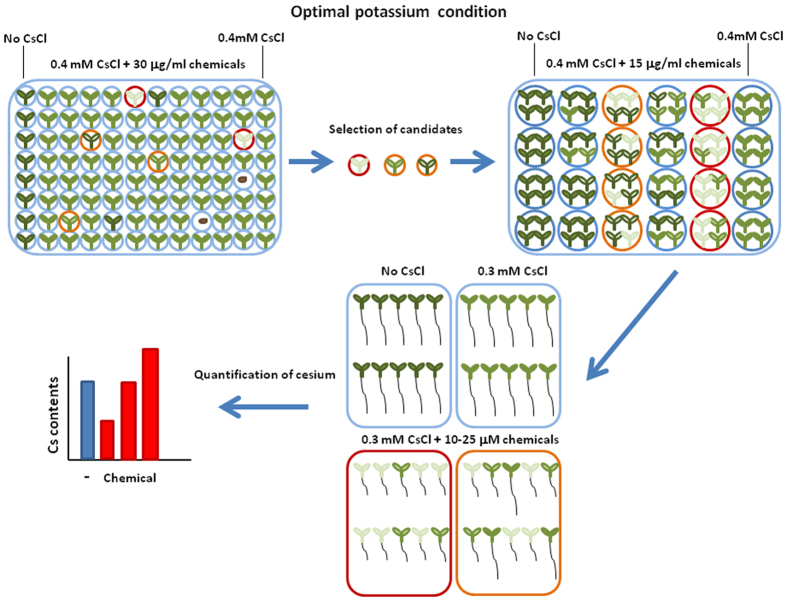
Schematic representation of chemical library screening. Wild type (Col-0) seeds were sown on media supplemented with indicated concentrations of cesium and the target chemicals under optimal (1.75 mM) potassium conditions and grown for 8 days. Those chemicals which conferred cesium-triggered phenotype to the plants were chosen as candidates. Further screenings were performed on a larger scale. Cesium concentrations in the seedlings were determined at the final screening.

In order to accelerate the process of cesium phytoaccumulation, suitable compounds for chemical application to promote cesium uptake were screened using a chemical library comprised of 10,000 small compounds (Fig. 1). A model plant, *Arabidopsis thaliana*, was analysed as it was suitable for testing a large number of seedlings in a small space in a short period of time. In optimal potassium conditions (1.75 mM K), 0.4 mM cesium hardly confers visible negative effects on plants. However, if a chemical increases cesium accumulation then the plants show cesium-triggered phenotype such as stunting and chlorosis^31^. Focusing on the cesium-triggered phenotype, chemicals were selected and narrowed down through second and third screening. Thirty-nine chemicals were selected for the visible phenotype after the third screening. In the fourth screening, to clarify whether the phenotype was actually triggered by high accumulation, cesium concentrations were quantified by an atomic absorption spectrometer and 14 out of 39 chemicals were confirmed as cesium accumulators in *Arabidopsis* (Table 1).

### Isolation of a cysteine derivative as a cesium accumulator

**Figure 2:**
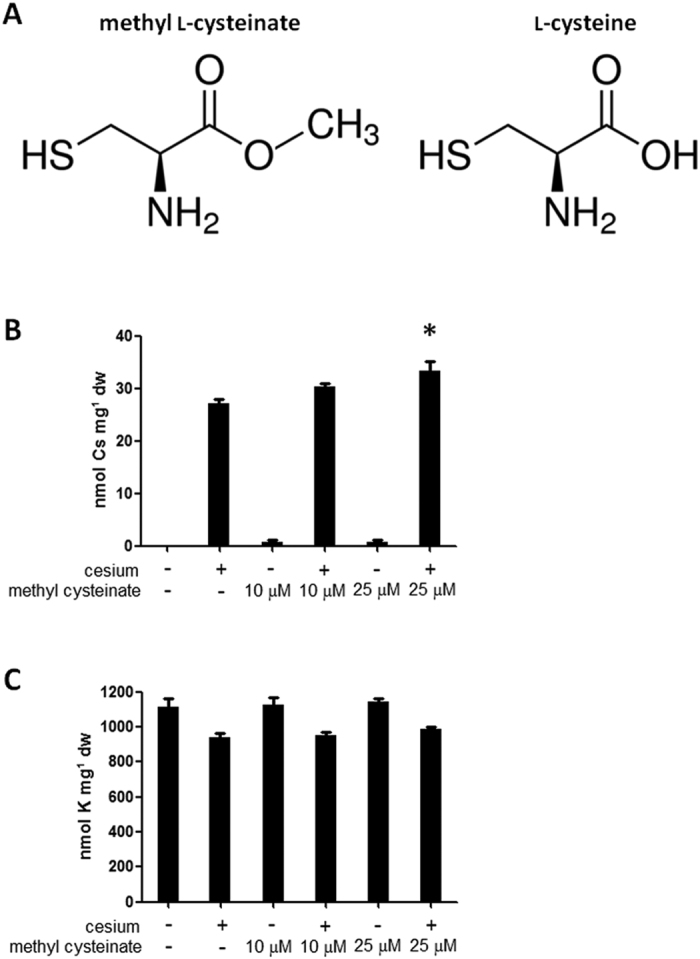
Effects of methyl cysteinate. (**A**) Chemical structures of methyl cysteinate and cysteine. (**B**) Cesium and (**C**) potassium concentrations in wild type (Col-0) seedlings grown under optimal (1.75 mM) potassium conditions in the presence or absence of 0.3 mM CsCl and the indicated concentrations of methyl cysteinate for 8 days. Error bars indicate standard error for three biological replicates and an asterisk indicates a statistically significant difference (*P* < 0.001) compared to cesium controls.

**Figure 3:**
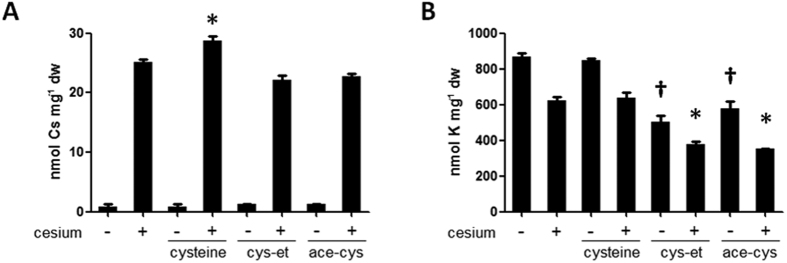
Effects of cysteine and its derivatives. (**A**) Cesium and (**B**) potassium concentrations in wild type (Col-0) seedlings grown under optimal (1.75 mM) potassium conditions in the presence or absence of 0.3 mM CsCl and 250 μM cysteine, cysteine ethyl ester (cys-et) or *N*-acetylcysteine (ace-cys) for 8 days. Error bars indicate standard error for three biological replicates and asterisks and daggers indicate statistically significant differences (*P* < 0.01) compared to cesium and no cesium controls, respectively.

One of the cesium accumulators selected was found to be a cysteine derivative, methyl cysteinate (C_4_H_9_NO_2_S, Table 1 #1 and Fig. 2A), where a hydrogen atom of the carboxyl group is replaced by a methyl group. Since an amino acid derivative can be speculated to readily function inside plants to exert a cesium accumulation effect or perhaps be involved in the primary metabolic pathway, methyl cysteinate was selected for further study. Methyl cysteinate at a concentration of 25 μM promotes increased cesium accumulation in plants by 22.4% (*P* < 0.001) compared to that in non-treated plants (Fig. 2B). It was observed that potassium concentrations were not altered by methyl cysteinate application (Fig. 2C). To confirm whether cysteine or other cysteine derivatives also helps plants accumulate cesium, cysteine, cysteine ethyl ester and *N*-acetylcysteine, were tested. Results from concentrations ranging from 10 to 1000 μM were analysed to determine the optimal functional concentration for each chemical (Supplement Fig. 1). Cysteine showed a cesium accumulation effect but at the lower extent (14.1% increase, *P* < 0.01) and at a much higher concentration (250 μM) compared to methyl cysteinate (Figs 2B and 3A). Application of cysteine ethyl ester and *N*-acetylcysteine did not increase cesium accumulation but severely stunted plants and dramatically decreased potassium concentrations (Fig. 3A,B). By contrast, cysteine did not reduce potassium concentrations in plants (Fig. 3B), consistent with what was observed for methyl cysteinate (Fig. 2C). Application of methyl cysteinate did not exhibit a cesium accumulation effect at 250 μM, probably because the plant growth was severely retarded at that concentration.

### Metabolite profiling analysis of plants treated with cesium and in potassium deficiency

**Figure 4:**
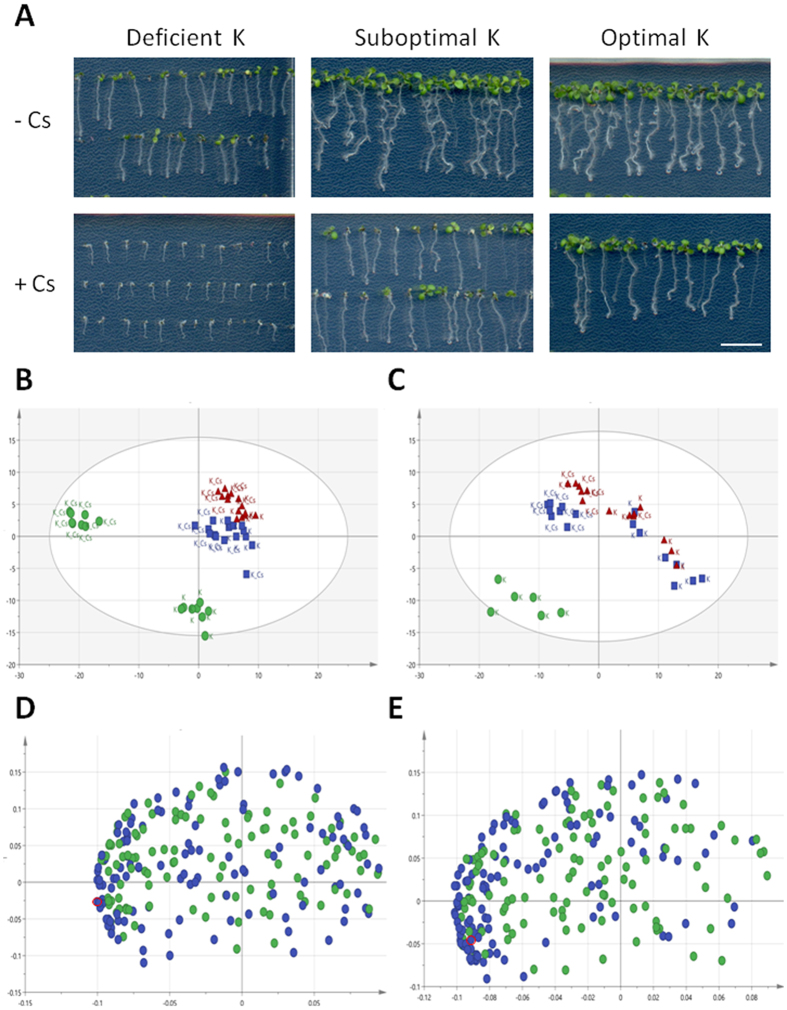
Metabolite profiling analysis. (**A**) Phenotype of wild type (Col-0) seedlings grown under optimal (1.75 mM), suboptimal (0.5 mM) or deficient (25 μM) potassium conditions in the presence or absence of 0.3 mM CsCl for 9 days. The scale bar denotes 1 cm. (**B**) Principal component (PC) analysis score scatter plot in the roots and (**C**) in the shoots. Red triangles, blue squares and green circles represent biological replicates (n = 7–8) from optimal, suboptimal and deficient potassium conditions (with and without cesium), respectively. PC1 (horizontal) and PC2 (vertical) account for 40.4% and 15.5%, respectively, in the roots and 39.3% and 15.5%, respectively, in the shoots. (**D**) PC analysis loading scatter plot in the roots and (**E**) in the shoots. Blue and green circles represent annotated and non-annotated peaks, respectively. Peaks for cysteine are highlighted in red.

Since contribution of cysteine was suggested in cesium accumulation, primary metabolites were investigated to reveal the metabolic shift that occurs in response to cesium treatment in plants. It is known that cesium and potassium ratios are important determinants of plant performance^31^, so optimal (1.75 mM), suboptimal (0.5 mM) and deficient (25 μM) potassium conditions in the presence and the absence of cesium were analysed (Fig. 4A). Seedlings grown in optimal and suboptimal potassium conditions were both healthy and almost indistinguishable from one another while growth of those in a deficient potassium condition were visibly stunted. The effects of cesium varied in accordance with the potassium conditions although the concentrations of cesium were constant: there was marginal growth retardation in the optimal potassium condition, clear root stunting and aerial chlorosis in the suboptimal potassium condition and extremely severe growth retardation in the deficient potassium condition. Principal component (PC) analysis resulted in samples from the optimal and suboptimal potassium conditions being clustered together and well-separated from those from the optimal potassium condition with cesium, the suboptimal potassium condition with cesium, the deficient potassium condition and the deficient potassium condition with cesium, in this order, along the PC1 (Fig. 4B,C). These results are in agreement with the severity of the growth phenotype (Fig. 4A) and suggest that growth retardation caused by cesium is not solely because of cesium-induced potassium deficiency in terms of primary metabolite composition in plants. Many amino acids and sugars increased in response to cesium including asparagine, glutamine, proline, glucose and sucrose possibly due to protein and carbohydrate degradation. By contrast, cesium-triggered shifts in other primary metabolites such as the participants in the tricarboxylic acid (TCA) cycle including citric acid and isocitric acid were less clear (Tables 2 and 3 - Table supplement 1–4). Cesium-treated plants showed higher levels of cysteine in the roots in suboptimal and deficient potassium conditions and in the shoots in optimal and suboptimal potassium conditions (shoot samples were missing for the deficient potassium condition as they were too small to obtain sufficient material). The loading scatter plots demonstrate that cysteine is one of the strongest driving factors of PC1 especially in the roots (Fig. 4D,E), which correlated with the dramatic increase of cysteine induced by cesium application in the deficient potassium condition (Table 2). Since cysteine is biosynthesised from serine and goes through interconversion with methionine, cesium accumulation ability driven by serine and methionine was assessed. However, neither showed any cesium accumulation effect at the concentration at which cysteine exerts its effect (250 μM), rather they stunted plant growth especially in the shoots for serine and in the roots for methionine.

### Mode of action of methyl cysteinate in cesium phytoaccumulation

**Figure 5:**
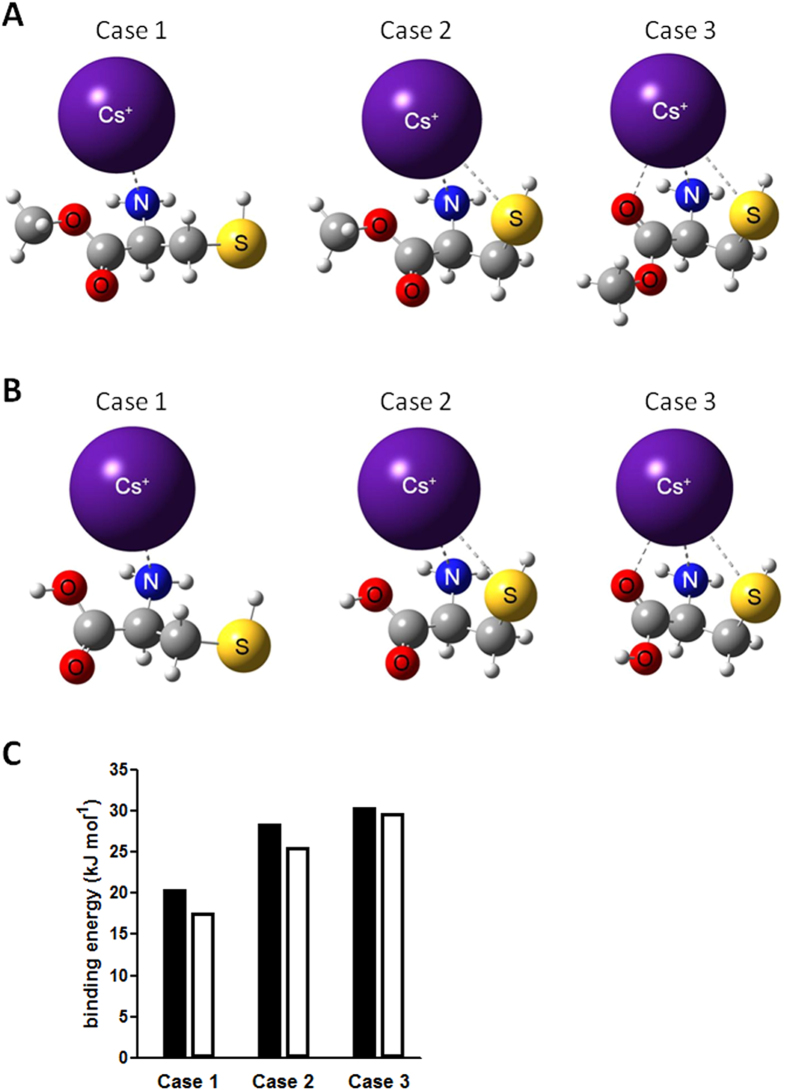
Quantum mechanical modelling for methyl cysteinate and cysteine. (**A**) Schematic representation of possible cesium binding structures for methyl cysteinate and (**B**) cysteine. Each atom is represented with van der Waals radii. (**C**) Binding energy of methyl cysteinate (black bars) and cysteine (white bars) with cesium for each case.

Methyl cysteinate (and cysteine to a lesser extent) promote cesium accumulation in *Arabidopsis*. In order to understand the mode of action, quantum mechanical calculations were performed. Three possible cases of cesium binding were predicted for each chemical being tested: Case 1, representing single interaction with the amino group, Case 2, representing chelation with the amino and sulphydryl groups, and Case 3, representing chelation with the amino, sulphydryl and carbonyl groups (Fig. 5A,B). Methyl cysteinate and cysteine were predicted to have high enough maximum binding energy with a cesium ion, 30.5 kJ mol^−1^ for methyl cysteinate and 29.7 kJ mol^−1^ for cysteine (Fig. 5C). The slightly lower binding energy in each case for cysteine relative to methyl cysteinate might explain the difference in the range of working concentrations for cesium accumulation. In addition, a difference in dipole moments for methyl cysteinate and cysteine, 1.34 D and 1.14 D, respectively, may contribute to the stability of each cesium complex.

**Figure 6:**
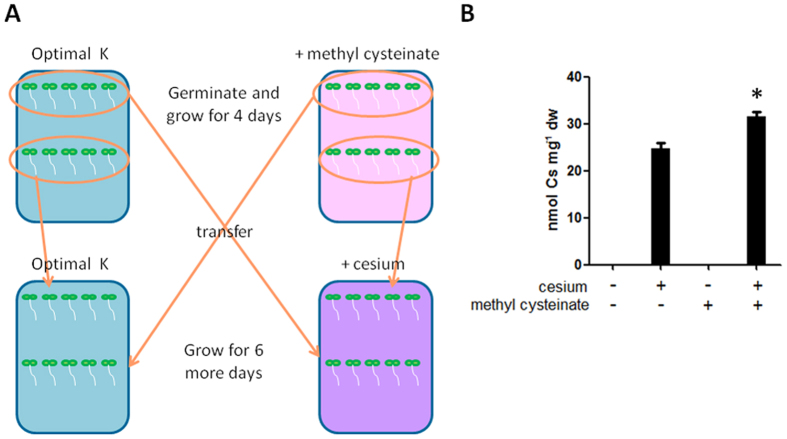
Effects of methyl cysteinate pre-treatment on cesium accumulation. (**A**) Schematic representation of transfer experiments. Wild type (Col-0) seedlings grown under optimal (1.75 mM) potassium conditions in the presence (pink plate) or absence (blue plate) of 25 μM methyl cysteinate for 4 days were transferred to fresh media containing optimal concentrations of potassium with (purple plate) or without (blue plate) 0.3 mM CsCl and grown for 6 more days prior to harvest. (**B**) Cesium concentrations in the plants. Error bars indicate standard error for three biological replicates and an asterisk indicates a statistically significant difference (*P* < 0.001) compared to cesium controls.

Furthermore, transfer experiments were performed to clarify the site of action of methyl cysteinate, whether within the plant, on the surface or outside of plant cells. First, wild type (Col-0) seeds of *Arabidopsis* were germinated on media containing optimal concentrations of potassium and 25 μM of methyl cysteinate. Seedlings were grown for 4 days and transferred to fresh media containing optimal concentrations of potassium and 0.3 mM CsCl. Seedlings were grown for 6 more days prior to harvest and cesium concentrations in plants were determined. In this system (Fig. 6A), cesium and methyl cysteinate never encounter each other except within the plant body. Methyl cysteinate exerted a cesium accumulation effect in the transfer system (Fig. 6B), suggesting that methyl cysteinate functions on the root surface or inside the plant cell.

## Discussion

Conventional techniques to extract metals from soil are both economically and environmentally challenging as well as costly. The use of plants for metal extraction has become a focus of attention since it is direct and environmentally benign as it relies on the ability of plants to absorb the metal of interest. In order to improve phytoaccumulation efficiency, various plant species and contributory factors have been investigated, whether for extracting valuable metals or removing contaminants from soil^1^. In our current study, chemical library screening was performed to isolate small organic compounds with potential to promote increased cesium accumulation in plants. Cesium, an alkali metal with a wide range of commercial applications, occurs relatively rarely in nature and a process for the recycling of the metal has not yet been established^3^. Moreover, radioactive isotopes such as cesium-134 and cesium-137 emitted from nuclear waste and accidents are highly toxic for humans and the ecosystem as a whole. Plants readily take up cesium as it has similar physicochemical properties as the essential nutrient potassium but, the efficiency of natural accumulation of cesium is not very high. Establishing the means for more efficient cesium phytoaccumulation would be beneficial both for resource recovery for a currently non-recyclable metal and for phytoremediation purposes. It is postulated that chemical applications could either convert the metal to a form more available for plant absorption or ‘re-programme’ the plant system to trigger increased accumulation of the metal. Naturally, chemical amendment is potentially applicable to a wide range of plant species and does not invoke concern on uncontrollable plant propagation as is the case with genetically modified plants. Out of 10,000 chemical compounds tested, 14 chemicals were isolated as cesium accumulators in *Arabidopsis thaliana* (Table 1). Of those, a cysteine derivative, methyl cysteinate, was found to accumulate 22.4% higher concentrations of cesium (Fig. 2B). Cysteine had a minor cesium accumulation effect but not the other derivatives such as cysteine ethyl ester and *N*-acetylcysteine or other amino acids which share the same metabolic pathway such as serine and methionine (Fig. 3A). These results suggest that it is the particular structure of methyl cysteinate that exerts this enhanced cesium accumulation effect rather than the cysteine metabolic pathway as a whole. Classic studies have reported that cysteine is capable of binding with a variety of metals including zinc, lead, copper, nickel and cobalt, yielding by far the highest stability constants of all the amino acids tested^32^,^33^. In the case of the zinc-cysteine complex, the sulphydryl and amino groups of cysteine have been suggested as the actual binding sites^33^, which corresponds with the high binding energy for Case 2 for methyl cysteinate and cysteine with cesium predicted from our calculations (Fig. 5C). Li and Manning have also indicated that for the zinc and lead complexes, methyl cysteinate is less stable compared to cysteine, in contrast to what we have observed in the case of cesium possibly due to the difference in ionic size. Methyl cysteinate was found to be more effective in improving cesium accumulation and worked at one order of magnitude lower concentrations compared to cysteine. This might be explained by the higher dipole moment and higher binding energy with cesium for methyl cysteinate (Fig. 5C). In the biosorption system using marine algal biomass, cysteine has been shown to increase gold-cyanide sorption when protonated^34^. Cysteine and *N*-acetylcysteine have been demonstrated to form complexes with silver in human lymphocytes and serve as a metal detoxification mechanism by inhibiting the incorporation of silver into a protein^35^. One of the most conserved proteins in evolution, ATP-binding cassette protein ABCE1, has a unique N-terminal region with eight conserved cysteine residues and these residues have been suggested as coordinating iron-sulphur clusters essential for protein function^36^. These findings, together with our findings, strongly suggest that cysteine and its derivatives have great potential in metal binding. Upon metabolite profiling analysis, cysteine was shown to increase in response to cesium treatment (Tables 2 and 3) and it was demonstrated as a major factor which separated the PC1 both in the roots and in the shoots (Fig. 4B–E). These results might imply that there is a positive feedback loop between cysteine concentrations and cesium accumulation in plants. It may be that cesium increases the levels of cysteine either via protein degradation or *de novo* synthesis which, in turn further promote cesium accumulation. It has previously been reported that a nickel hyperaccumulator species of *Alyssum* shows enhanced production of another amino acid histidine in response to the presence of nickel^37^. These authors have also shown that exogenous application of histidine to a non-accumulating species promotes nickel tolerance as well as nickel transport to the shoots. In our case, the results from the transfer experiments support this notion and indicate that methyl cysteinate absorbed by plants, without directly encountering cesium in the growth medium, could exert a cesium accumulation effect (Fig. 6B), suggesting that binding of methyl cysteinate and cesium occurs either on the surface or inside of the plant cells. It is tempting to speculate that cysteine accumulates in the plant body in response to exogenous cesium and is synthesised into methyl cysteinate which binds with cesium to protect plants from its deleterious effects but further experimental support is required to confirm this point. Although both methyl cysteinate and cysteine showed higher binding energy with potassium than with cesium, they did not increase potassium concentrations in plants, suggesting the existence of different regulatory mechanisms in play to distinguish the two cations.

Taken together, our results indicate that methyl cysteinate improves cesium phytoaccumulation efficiency through as-yet-unknown mechanism but which could be through promoting cesium adhesion to the root surface or inhibiting cesium extrusion from the plant cells due to a direct binding with cesium. Moreover, the PC analysis of primary metabolites demonstrated the distinct metabolic changes that occur in response to cesium treatment and to potassium deficiency (Fig. 4B,C), suggesting that potassium deficiency caused by cesium is not the major factor in cesium-induced growth inhibition in plants as it has long been believed, but may be direct cesium toxicity. The results also highlight the importance of potassium concentrations and in turn the cesium/potassium ratios for plant performance in terms of metabolite profiles. This point is particularly noteworthy as plants under optimal or suboptimal potassium conditions in the absence of cesium clustered together in the PC analysis (Fig. 4B,C) and their phenotype was nearly indistinguishable (Fig. 4A). Further investigation of individual metabolites which are responsive to cesium and potassium deficiency, together with functional analysis of the other 13 cesium accumulator chemicals that have been identified might provide some clues to a more precise molecular understanding of cesium/potassium uptake and response in plants.

## Methods

### Plant material and growth conditions

The *Arabidopsis thaliana* (L.) Heynh. accession wild type Col-0 was used. Seeds were surface-sterilised with 70% (v/v) ethanol and 0.05% (v/v) Triton X-100 and sown on media containing 2 mM Ca(NO_3_)_2_, 0.5 mM phosphoric acid, 0.75 mM MgSO_4_, 50 μM H_3_BO_3_, 10 μM MnCl_2_, 2 μM ZnSO_4_, 1.5 μM CuSO_4_, 0.075 μM NH_4_Mo_7_O_24_ and 74 μM Fe-EDTA, pH 5.8, with Ca(OH)_2_, 1% (w/v) sucrose and 0.6% (w/v, for the screening and metabolite profiling analysis) or 1% (w/v, for the rest of the experiments) of SeaKem agarose (Lonza, Basel, Switzerland) supplemented with designated concentrations of KCl, CsCl and other chemicals. After stratification for 3 to 4 days at 4 °C, plants were placed in a growth cabinet at 22 °C in a 16 h light/8 h dark photocycle with a light intensity of 70–90 μmol m^−2^ sec^−1^.

### Chemical library screening

Small synthetic organic compounds from the Enamine (http://www.enamine.net/) chemical library dissolved in dimethylsulfoxide (DMSO) as 3 mg ml^−1^ stock solution were used. Five to six Col-0 seeds were sown in each well containing 100 μl of agar-based growth media containing optimal (1.75 mM) KCl, 0.4 mM CsCl and 30 μg ml^−1^ chemical in a 96-well plate. Each plate hosted cesium controls (0.4 mM CsCl with no chemical added) and no cesium controls (no CsCl added) containing equivalent volume (1 μl) of DMSO. Seedlings were grown for 8 days and compared to those on cesium and no cesium controls. The selected candidate compounds were investigated further and narrowed down through second, third and fourth screening. The second screening was performed with three replicates for each chemical at the same scale as in the initial screening. The third screening, two replicates for each chemical, was performed using 24-well plates with each well containing 1.5 ml of growth media (1.75 mM KCl with or without 0.4 mM CsCl), 15 μg ml^−1^ chemical and 10 seeds. The fourth screening (three replicates) was performed using petri dishes containing 50 ml of growth media (1.75 mM KCl with or without 0.3 mM CsCl), 10 and 25 μM chemical, 40–50 seeds. Eight-day-old seedlings were harvested and cesium and potassium concentrations in the plants were determined.

### Elemental analysis

Seedlings were washed in Milli-Q water, dried on a paper towel, placed in a paper envelope and dried in an oven at 65 °C for 3–4 days. Approximately 2 mg of dried samples were extracted in 1 ml of 60% (v/v) HNO_3_ at 125 °C for 1 hour, followed by 1 ml of 30% (v/v) H_2_O_2_ and diluted with Milli-Q water to get a total volume of 10 ml. For potassium analysis, samples were further diluted 10 times with 6% (v/v) HNO_3_. For cesium analysis, 0.1% (w/v) KCl was added to each sample and standard solution to prevent ionisation of cesium, according to the manufacturer’s instructions (PerkinElmer, Waltham, Massachusetts). Potassium and cesium concentrations were measured on a flame atomic absorption spectrometer AAnalyst 200 (PerkinElmer). Concentrations were calculated against each standard curve, and one-way ANOVA with Bonferroni’s multiple comparison posttest was performed using Prism (GraphPad Software, La Jolla, California) to determine the statistical significance.

### Metabolite profiling analysis

Col-0 seeds were sown on media containing optimal (1.75 mM), suboptimal (0.5 mM) or deficient (25 μM) KCl with or without 0.3 mM CsCl and grown for 9 days. Roots and shoots were harvested separately except in the case of 25 μM KCl + 0.3 mM CsCl where only roots were harvested. Samples were washed in Milli-Q water, dried on a paper towel and flash frozen in liquid N_2_ to quench enzymatic activity (7–8 biological replicates). Gas chromatography – time-of-flight – mass spectrometry (GC-MS) analysis was performed as described previously^38^ with slight modification. A total of 0.5 mg fresh weight of samples were subjected to derivatisation. An equivalent of 0.6 μg of the derivatised sample was injected into the GC-MS instrument. The chromatograms were pre-processed and normalised as described previously^39^,^40^. All chemicals, except the isotope-labelled chemicals used for GC-MS analysis^38^, were purchased from Wako (Tokyo, Japan) or Sigma-Aldrich (St. Louis, Missouri).

### Theoretical modelling

To evaluate the binding energy of each chemical, quantum mechanical calculations were performed. First, the geometry of each chemical was optimised using the density functional theory (DFT) method with the B3LYP hybrid exchange-correlation energy functional^41^,^42^ and 6–31 G** level of basis set. After optimisation, an electrostatic potential (ESP) map was calculated to find more probable ion affinitive sites. One to four positive ion attractive sites for each chemical were found and each ion was placed at every site one at a time to model the initial conformation of the chemical-ion complex. From the structure, geometry optimisation was performed with the DFT method with the B3LYP hybrid exchange-correlation energy functional^41^,^42^ and the atom-centred LanL2DZ basis set^43^,^44^ for the heavy cesium ion. The polarisable continuum model (PCM) with the integral equation formalism variant^45^ was used to describe the solvation with water. Grimme’s dispersion correction (DFT-D)^46^,^47^ was used to model dispersion interactions. After the optimisation, the binding energy, *E*_*bind*_, was calculated using the definition below.


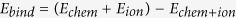


where *E*_*chem*+*ion*_ is the total energy of the ion-chemical complex, *E*_*chem*_ and *E*_*ion*_ are the energy of a chemical and an ion in vacuum, respectively. The ion affinities of cesium and potassium were calculated for each site and the maximum *E*_*bind*_ for each ion was chosen. All optimised structures were converged and found to have no negative frequencies. The dipole moments were calculated with Hirshfeld charge^48^,^49^,^50^. All quantum calculations were calculated by the commercial software, Gaussian 09 Rev. D (http://www.gaussian.com/g_tech/g_ur/m_citation.htm).

## Additional Information

**How to cite this article**: Adams, E. *et al*. A novel role for methyl cysteinate, a cysteine derivative, in cesium accumulation in *Arabidopsis thaliana. Sci. Rep.* **7**, 43170; doi: 10.1038/srep43170 (2017).

**Publisher's note:** Springer Nature remains neutral with regard to jurisdictional claims in published maps and institutional affiliations.

## Supplementary Material

Supplementary Data

## Figures and Tables

**Table 1:** Chemical formula for the cesium accumulators isolated through screening, maximum percentage increase in cesium concentrations in plants and the concentration of the chemical used to reach the maximum percentage increase.

number	chemical formula	% increase of cesium	chemical concentration
1	C_4_H_9_NO_2_S	22.4	25 μM
2	C_9_H_8_N_2_OS_2_	74.5	25 μM
3	C_14_H_14_O_5_	45.4	50 μM
4	C_13_H_14_N_2_O_2_S_2_	49.6	10 μM
5	C_13_H_15_N_5_	51.5	25 μM
6	C_27_H_18_BrN_3_O	43.7	25 μM
7	C_22_H_22_N_4_O_3_S_3_	17.1	10 μM
8	C_10_H_10_F_2_N_4_S	29.3	10 μM
9	C_16_H_11_ClO_4_	40.1	25 μM
10	C_11_H_15_NO_5_S_2_	11.5	10 μM
11	C_21_H_15_F_3_N_2_	14.7	25 μM
12	C_18_H_14_FN_3_O_4_	21.4	25 μM
13	C_8_H_7_N_3_O_4_	17.0	25 μM
14	C_21_H_20_N_2_O_9_	25.0	10 μM

**Table 2:** Amino acid levels in the roots of Col-0 treated with potassium deficiency (−K), suboptimal potassium (0.5 K), optimal potassium (1.75 K) with or without cesium (Cs).

amino acid	−K	−K + Cs	0.5 K	0.5 K + Cs	1.75 K	1.75 K + Cs
Alanine	133 (0.66)	2220 (81.8)	265 (27.1)	494 (59.8)	443 (33.3)	545 (50.2)
Asparagine	15.6 (1.58)	150.7 (15.5)	9.92 (1.15)	20.2 (2.91)	6.56 (0.56)	10.0 (1.76)
Aspartic acid	5.28 (0.28)	26.0 (3.13)	41.9 (2.87)	34.1 (2.71)	71.4 (4.32)	80.1 (5.94)
Cysteine	7.97 (0.68)	134 (12.6)	0.82 (0.08)	2.45 (0.35)	1.19 (0.20)	1.22 (0.08)
Glutamic acid	5.72 (0.37)	18.7 (2.04)	26.2 (1.55)	24.3 (1.98)	40.8 (2.41)	36.8 (1.38)
Glutamine	18400 (2550)	49400 (5310)	1990 (141)	6220 (680)	1890 (122)	1860 (190)
Glycine	10.6 (0.81)	203 (11.7)	10.5 (0.80)	9.48 (0.96)	12.3 (0.61)	10.8 (1.30)
Histidine	1.33 (0.18)	9.09 (0.60)	0.50 (0.05)	0.79 (0.10)	0.22 (0.02)	0.42 (0.06)
Isoleucine	216 (19.0)	190 (13.8)	182 (23.1)	52.0 (6.68)	33.8 (4.21)	74.6 (14.2)
Leucine	457 (21.6)	752 (49.5)	560 (46.0)	184 (20.4)	159 (17.6)	265 (31.1)
Lysine	15.3 (1.07)	41.2 (1.61)	10.4 (0.93)	6.27 (0.58)	2.30 (0.13)	6.10 (0.79)
Methionine	2250 (69.8)	3480 (96.3)	4420 (166)	3720 (105)	5680 (136)	5550 (58.6)
Phenylalanine	21.8 (1.70)	43.3 (5.14)	42.0 (2.89)	44.3 (5.47)	20.4 (0.98)	28.1 (2.23)
Proline	32.4 (2.54)	621 (37.5)	22.4 (1.49)	40.1 (10.1)	24.6 (1.45)	23.0 (3.09)
Serine	727 (46.4)	4880 (554)	1150 (100)	2050 (293)	1530 (100)	1300 (112)
Threonine	1320 (85.0)	2130 (253)	1200 (105)	3440 (355)	829 (49.7)	1410 (89.4)
Tryptophan	0.78 (0.06)	0.71 (0.04)	0.85 (0.10)	0.35 (0.05)	0.21 (0.02)	0.45 (0.09)
Tyrosine	15.9 (1.91)	34.3 (1.89)	54.9 (5.66)	17.8 (2.14)	11.1 (1.20)	26.5 (4.51)
Valine	547 (36.0)	994 (53.7)	369 (37.1)	255 (25.9)	149 (7.83)	230 (30.0)

Values are the mean intensities of the signals from seven to eight biological replicates with standard errors in parentheses.

**Table 3:** Amino acid levels in the shoots of Col-0 treated with potassium deficiency (−K), suboptimal potassium (0.5 K), optimal potassium (1.75 K) with or without cesium (Cs).

amino acid	−K	0.5 K	0.5 K + Cs	1.75 K	1.75 K + Cs
Alanine	78.2 (11.4)	145 (20.3)	1400 (135)	235 (36.3)	848 (71.5)
Asparagine	27.5 (3.61)	4.39 (0.73)	10.4 (0.80)	1.14 (0.14)	4.79 (0.38)
Aspartic acid	6.64 (0.59)	48.8 (5.32)	45.1 (1.98)	63.3 (8.55)	92.1 (7.98)
Cysteine	6.92 (1.06)	0.43 (0.07)	1.42 (0.14)	0.66 (0.08)	1.02 (0.06)
Glutamic acid	6.30 (0.75)	22.3 (3.00)	29.7 (1.63)	18.9 (2.07)	41.7 (3.61)
Glutamine	29800 (3630)	631 (59.0)	4690 (413)	853 (87.9)	2440 (288)
Glycine	79.9 (16.6)	6.21 (1.01)	17.7 (2.19)	15.6 (1.73)	35.0 (8.17)
Histidine	0.94 (0.17)	0.03 (0.00)	0.11 (0.01)	0.02 (0.00)	0.05 (0.00)
Isoleucine	217 (48.4)	1.06 (0.17)	4.36 (0.53)	1.09 (0.25)	4.44 (0.74)
Leucine	256 (48.7)	15.3 (3.34)	31.5 (1.89)	10.1 (2.27)	27.8 (2.91)
Lysine	7.34 (1.32)	0.44 (0.07)	1.22 (0.08)	0.27 (0.03)	0.71 (0.06)
Methionine	2470 (118)	2970 (207)	4110 (124)	4400 (355)	5630 (389)
Phenylalanine	154 (33.0)	8.88 (1.32)	31.4 (1.77)	5.98 (1.01)	15.5 (1.19)
Proline	8.71 (1.08)	5.50 (0.59)	64.1 (5.09)	5.09 (0.53)	25.5 (3.75)
Serine	3730 (750)	176 (21.0)	3640 (298)	3030 (631)	3720 (377)
Threonine	1830 (292)	85.5 (13.9)	490 (34.1)	154 (27.1)	444 (42.5)
Tryptophan	2.43 (0.49)	0.03 (0.00)	0.12 (0.01)	0.03 (0.00)	0.08 (0.01)
Tyrosine	22.2 (3.97)	1.22 (0.26)	2.23 (0.18)	0.71 (0.17)	1.76 (0.24)
Valine	562 (96.6)	26.5 (3.82)	101 (4.66)	26.0 (3.10)	71.1 (6.18)

Values are the mean intensities of the signals from seven to eight biological replicates with standard errors in parentheses. Shoot samples were missing for the deficient potassium condition with cesium as they were too small to obtain sufficient material.
