# Supplementary material for: A novel role for methyl cysteinate, a cysteine derivative, in cesium accumulation in Arabidopsis thaliana
Source: Sci Rep. 2017 Feb 23;7:43170. doi: 10.1038/srep43170 (PMC5322390; doi:10.1038/srep43170)
Supplement: Supplementary Data [file srep43170-s1.docx]

**
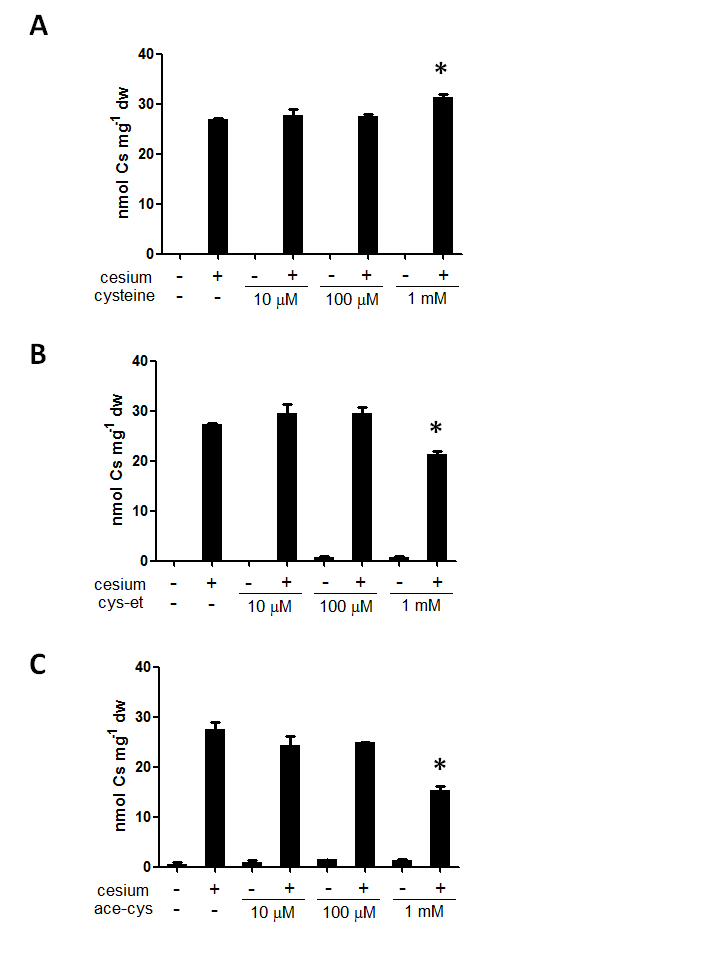
**

**Supplementary Fig. 1. Functional concentration range test for cysteinate and its derivatives.**

(**A**) Cesium concentrations in wild type (Col-0) seedlings grown under optimal (1.75 mM) potassium conditions in the presence or absence of 0.3 mM CsCl and the indicated concentrations of cysteine, (**B**) cysteine ethyl ester (cys-et) and (**C**) *N*-acetylcysteine (ace-cys) for 8 days. Error bars indicate standard error for three biological replicates and an asterisk indicates a statistically significant difference (*P* < 0.001) compared to cesium controls.

**Table supplement 1.**

Sugar levels in the roots of Col-0 treated with potassium deficiency (-K), suboptimal potassium (0.5K), optimal potassium (1.75K) with or without cesium (Cs). Values are the mean intensities of the signals from seven to eight biological replicates with standard errors in parentheses.

| amino acid | -K | -K + Cs | 0.5K | 0.5K + Cs | 1.75K | 1.75K + Cs |
| --- | --- | --- | --- | --- | --- | --- |
| Arabinose | 21.9 (1.71) | 8.06 (1.14) | 21.8 (1.34) | 18.3  (1.85) | 16.8 (0.66) | 19.9  (0.89) |
| Fructose | 11600 (1200) | 35200 (1450) | 3390 (263) | 4660  (689) | 3580 (337) | 2460  (164) |
| Fructose-6-P | 11.3 (0.85) | 33.1 (3.01) | 33.1 (1.74) | 30.1  (2.96) | 21.3 (0.82) | 33.1  (1.84) |
| Glucose | 336 (14.9) | 963 (28.6) | 93.6 (8.99) | 107  (12.9) | 101 (6.87) | 75.8  (1.84) |
| Glucose-6-P | 18.5 (1.55) | 75.1 (9.85) | 62.7 (4.01) | 57.9  (6.14) | 49.5 (2.82) | 73.9  (4.80) |
| Maltose | 1.35 (0.31) | 6.53 (1.04) | 1.45 (0.14) | 1.29  (0.22) | 1.43 (0.24) | 1.81  (0.19) |
| Mannose | 138 (13.2) | 20.4 (2.27) | 27.3 (2.98) | 0.50  (0.16) | 38.4 (3.83) | 19.7  (2.82) |
| Raffinose | 11.7 (1.26) | 3.97 (0.60) | 0.39 (0.11) | 4.24  (1.24) | 0.46 (0.11) | 1.16  (0.14) |
| Ribose | 301 (27.5) | 2430 (194) | 246 (24.0) | 365  (46.0) | 185 (9.52) | 241  (28.8) |
| Sucrose | 51.0 (2.64) | 257 (4.25) | 11.8 (0.68) | 12.0  (1.90) | 12.1 (1.9) | 4.35  (0.68) |
| Trehalose | 14.6 (1.14) | 13.5 (1.80) | 28.7 (2.50) | 10.7  (1.28) | 44.3 (2.77) | 34.0  (2.75) |
| Xylose | 33.2 (3.53) | 182 (12.7) | 46.8 (4.59) | 11.8  (1.27) | 34.1 (1.76) | 27.4  (3.53) |

**Table supplement 2.**

Sugar levels in the shoots of Col-0 treated with potassium deficiency (-K), suboptimal potassium (0.5K), optimal potassium (1.75K) with or without cesium (Cs). Values are the mean intensities of the signals from seven to eight biological replicates with standard errors in parentheses. Shoot samples were missing for the deficient potassium condition as they were too small to obtain sufficient material.

| amino acid | -K | 0.5K | 0.5K + Cs | 1.75K | 1.75K + Cs |
| --- | --- | --- | --- | --- | --- |
| Arabinose | 50.6 (11.7) | 12.2 (1.75) | 18.2  (1.38) | 12.0  (1.38) | 17.1  (1.65) |
| Fructose | 25400 (1870) | 1960 (166) | 11800 (794) | 2420  (295) | 5010  (975) |
| Fructose-6-P | 27.1 (3.73) | 8.09 (0.99) | 19.6  (0.94) | 9.28  (1.25) | 17.9  (1.68) |
| Glucose | 432  (39.0) | 27.8 (2.57) | 141  (5.83) | 26.6  (2.74) | 63.0  (12.6) |
| Glucose-6-P | 7.58 (1.12) | 4.00 (0.64) | 21.4  (0.95) | 5.60  (0.63) | 11.1  (1.40) |
| Maltose | 0.11 (0.03) | 0.09 (0.02) | 0.24  (0.04) | 0.06  (0.02) | 0.14  (0.03) |
| Mannose | 18.2 (4.18) | 0.54 (0.15) | 6.04  (0.66) | 0.79  (0.54) | 1.78  (0.30) |
| Raffinose | 1.07 (0.12) | 0.12 (0.04) | 0.33  (0.09) | 0.14  (0.02) | 0.16  (0.03) |
| Ribose | 431  (58.0) | 71.0  (11.8) | 211  (14.6) | 26.8  (3.60) | 108  (10.7) |
| Sucrose | 86.6 (10.1) | 11.0  (1.62) | 69.7  (3.25) | 11.4  (2.24) | 33.6  (4.47) |
| Trehalose | 38.3 (6.67) | 5.63 (1.03) | 16.5  (0.94) | 8.20  (1.44) | 13.9  (1.46) |
| Xylose | 34.8 (5.15) | 8.62 (1.29) | 11.4  (1.04) | 12.2  (1.48) | 13.2  (1.29) |

**Table supplement 3.**

Other metabolite levels in the roots of Col-0 treated with potassium deficiency (-K), suboptimal potassium (0.5K), optimal potassium (1.75K) with or without cesium (Cs). Values are the mean intensities of the signals from seven to eight biological replicates with standard errors in parentheses.

| amino acid | -K | -K + Cs | 0.5K | 0.5K + Cs | 1.75K | 1.75K + Cs |
| --- | --- | --- | --- | --- | --- | --- |
| Citric acid | 344 (34.9) | 414 (30.2) | 18200 (877) | 17500 (943) | 24100 (1570) | 20000  (607) |
| Isocitric acid | 0.03 (0.00) | 0.03 (0.00) | 1.84 (0.11) | 1.90  (0.11) | 2.90 (0.09) | 2.42  (0.10) |
| 2-Oxoglutaric acid | 0.21 (0.05) | 10.3 (3.05) | 15.7 (1.80) | 5.82 (0.76) | 58.2 (4.22) | 24.0  (1.08) |
| Succinic acid | 0.05 (0.00) | 1.36 (0.15) | 0.61 (0.04) | 0.18 (0.02) | 2.72 (0.28) | 0.71  (0.08) |
| Malic acid | 707 (79.6) | 2940 (247) | 17000 (1130) | 6210 (489) | 27900 (1150) | 10300  (379) |
| Urea | 7.49 (0.72) | 1.67 (0.22) | 7.05 (1.37) | 2.23 (1.25) | 4.69 (0.33) | 4.83  (1.65) |
| Ornithine | 9.54 (1.00) | 109 (15.3) | 7.42 (1.12) | 10.9 (1.50) | 2.34 (0.34) | 4.60  (0.20) |
| GABA | 8380 (937) | 33400 (3380) | 1450 (156) | 4010 (622) | 1680 (192) | 1020  (174) |
| Spermidine | 35.9 (2.05) | 89.6 (9.68) | 41.5 (2.97) | 45.0 (4.66) | 36.4 (1.64) | 51.8  (1.80) |
| 1,3-Diaminopropane | 10.6 (0.81) | 24.4 (3.59) | 2.17 (0.13) | 5.44 (0.72) | 1.85 (0.07) | 2.42  (0.25) |
| Lignoceric acid | 1.14 (0.17) | 4.74 (0.26) | 2.75 (0.18) | 1.75 (0.20) | 2.87 (0.17) | 2.65  (0.14) |
| Palmitic acid | 1.50 (0.06) | 3.39 (0.13) | 1.59 (0.08) | 1.48 (0.07) | 1.66 (0.08) | 1.94  (0.07) |
| α-Linolenic acid | 101 (14.1) | 91.2 (8.85) | 95.3 (15.6) | 77.8 (10.6) | 109 (8.78) | 94.1  (9.06) |
| Inositol | 2410 (202) | 328 (33.6) | 853 (77.8) | 351  (42.3) | 770 (23.3) | 824  (55.0) |
| Inositol-1-phosphate | 2.68 (0.30) | 11.9 (1.51) | 7.70 (0.71) | 8.07 (0.94) | 10.5 (0.48) | 14.6  (0.75) |
| Homoserine | 0.22 (0.07) | 2.39 (0.88) | 0.04 (0.01) | 0.85 (0.19) | 0.29 (0.06) | 0.15  (0.02) |
| Shikimic acid | 15.0 (1.29) | 47.4 (3.74) | 15.8 (1.00) | 16.4 (1.50) | 30.9 (1.65) | 19.5  (0.82) |
| Suberic acid | 10.3 (9.12) | 19.0 (3.48) | 3.70 (1.45) | 4.59 (1.46) | 3.25 (0.70) | 6.08  (1.39) |
| Phosphoric acid | 1590 (75.6) | 3960 (270) | 2660 (202) | 4030 (178) | 2330 (92.5) | 3250  (163) |

**Table supplement 4.**

Other metabolite levels in the shoots of Col-0 treated with potassium deficiency (-K), suboptimal potassium (0.5K), optimal potassium (1.75K) with or without cesium (Cs). Values are the mean intensities of the signals from seven to eight biological replicates with standard errors in parentheses. Shoot samples were missing for the deficient potassium condition as they were too small to obtain sufficient material.

| amino acid | -K | 0.5K | 0.5K + Cs | 1.75K | 1.75K + Cs |
| --- | --- | --- | --- | --- | --- |
| Citric acid | 8580 (1050) | 4260 (239) | 10600 (708) | 5470  (702) | 9800  (961) |
| Isocitric acid | 0.99  (0.13) | 0.36 (0.02) | 0.91  (0.05) | 0.46  (0.05) | 0.82  (0.07) |
| 2-Oxoglutaric acid | 14.7  (2.22) | 15.4 (1.39) | 16.4  (0.77) | 25.7  (4.37) | 30.7  (4.23) |
| Succinic acid | 2.46  (0.34) | 1.81 (0.16) | 1.85  (0.07) | 2.22  (0.20) | 2.90  (0.23) |
| Malic acid | 48100 (4930) | 16400 (1180) | 19900 (960) | 22800 (2320) | 22300 (1470) |
| Urea | 0.79  (0.19) | 1.28 (0.36) | 1.26  (0.15) | 1.30  (0.22) | 1.47  (0.15) |
| Ornithine | 27.0  (10.8) | 1.61 (0.41) | 13.3  (1.64) | 3.66  (1.14) | 12.1  (1.79) |
| GABA | 7520  (743) | 420  (126) | 2410  (273) | 284  (85.1) | 1860  (276) |
| Spermidine | 41.4  (6.32) | 13.8 (2.13) | 32.8  (1.88) | 19.1  (2.77) | 29.0  (2.40) |
| 1,3-Diaminopropane | 13.8  (1.74) | 1.18 (0.14) | 5.24  (0.33) | 1.21  (0.13) | 4.10  (0.43) |
| Lignoceric acid | 0.92  (0.14) | 1.18  (0.11) | 1.61  (0.13) | 1.42  (0.14) | 1.95  (0.13) |
| Palmitic acid | 1.17  (0.05) | 1.13 (0.06) | 1.54  (0.08) | 1.24  (0.12) | 1.62  (0.07) |
| α-Linolenic acid | 98.4  (9.99) | 57.9 (6.55) | 82.2  (12.5) | 83.0  (13.3) | 119  (20.6) |
| Inositol | 103  (16.8) | 110  (27.2) | 196  (18.4) | 183  (27.9) | 273  (39.3) |
| Inositol-1-phosphate | 1.54  (0.25) | 2.14 (0.19) | 2.28  (0.29) | 2.59  (0.52) | 2.75  (0.33) |
| Homoserine | 2.12  (0.52) | 0.20 (0.07) | 1.70  (0.30) | 0.55  (0.10) | 2.16  (0.50) |
| Shikimic acid | 273  (107) | 9.45 (1.21) | 27.8  (2.91) | 14.8  (2.92) | 27.2  (2.64) |
| Suberic acid | 21.9  (16.8) | 13.6 (5.44) | 26.8  (11.7) | 27.4  (11.6) | 27.5  (14.2) |
| Phosphoric acid | 421  (23.5) | 588  (91.0) | 803  (59.5) | 450  (39.2) | 820  (91.7) |
